# Variation in Expression of Reference Genes across Life Stages of a Bee, *Megachile rotundata*

**DOI:** 10.3390/insects12010036

**Published:** 2021-01-06

**Authors:** Junhuan Xu, Dennis L. Welker, Rosalind R. James

**Affiliations:** 1 Department of Biology, Utah State University, 5305 Old Main Hill, Logan, UT 84322, USA; Junhuanxu@yahoo.com (J.X.); dennis.welker@usu.edu (D.L.W.); 2 Department of Plant Pathology, Ohio State University, 023 Selby Hall, 1680 Madison Ave., Wooster, OH 44691, USA; 3 USDA Agricultural Research Service, Pollinating Insects Research Unit, Logan, UT 84322, USA

**Keywords:** bees, diapause, diapausing, gene expression, *Megachile*, RT-qPCR, reference genes

## Abstract

**Simple Summary:**

Reference genes are key to normalizing expression data across samples of organisms collected after different treatments are applied, but often, reference genes are not properly validated for this purpose. In this report, we screened several genes for a solitary bee, *Megachile rotundata*, and identified two (RPS18, and RPL8) with very stable expression levels across all life stages of the bee, and under a variety of environmental conditions, including during and after diapause. These genes should make good reference genes. We also identified other genes with stable expression, even if used only for a limited number of developmental stages. This information is important for future gene expression studies on these bees, but it also demonstrates the importance of validating reference genes in general.

**Abstract:**

The alfalfa leafcutting bee, *Megachile rotundata* is widely used in the western United States as a pollinator for alfalfa seed production. Unfortunately, immatures experience high mortality in agriculturally managed populations. Quantified gene expression could be used to identify how this bee responds during different life stages to pathogens, environmental toxins, and other stresses, but stably expressed reference genes are needed to normalize transcription data. We evaluated twelve candidate genes for their transcription stability across different life stages, including during and after diapause. RPS18 and RPL8 were the two most stably expressed genes, followed by RPS5 and RPL27A. These genes were also very stable even during and after diapause, while the most variable genes being APN, PMIIM, NPC2, and Cr-PII had increased expression levels during larval growth and were also variable during and after diapause. The four reference genes we identified in *M. rotundata* may prove useful for transcriptomic studies in other bees as well, such as honey bees.

## 1. Introduction

In North America, the alfalfa leafcutting bee, *Megachile rotundata,* is a critical pollinator for alfalfa seed production [1,2]. However, bee health is frequently threatened by outside environmental elements, including exposure to pathogens, pesticides, and unfavorable weather conditions [3,4,5]. Gene expression analyses provide insights into the physiological mechanisms by which bees deal with these stresses [6,7]. Several immune response genes, detoxification genes and some signaling pathway systems have been identified for *M. rotundata* [8]; however, gene expression levels during different developmental stages are largely unknown. 

Northern blots, microarrays, and real-time, quantitative, reverse transcription polymerase chain reactions (RT-qPCR) were all used to quantify gene expression in living organisms. These methods can be very sensitive, especially RT-qPCR, but they typically require internal gene expression standards if expression levels are compared across treatments and experiments [9,10]. Typically, the reference genes used as internal standards are associated with proteins involved in basic, ubiquitous cellular functions, as these are most likely to have consistent levels of expression under most conditions.

Moderate transcript abundance and low variation in abundance in different individuals and life stages are important criteria for reference genes [11]. The 18S and 28S rRNAs were thought to be good reference genes for most animals [11,12], since they are abundant and have uniform expression levels in different tissues. However, a major drawback to using rRNA as a normalizer in RT-qPCR reactions is its lack of poly-A, which precludes its isolation in oligo(dT) based procedures [12]. Thus, we focused on genes transcribed by RNA polymerase II whose transcripts can be isolated using oligo(dT), such as the genes associated with glyceraldehyde 3-phosphate dehydrogenase (GAPDH), beta-actin (ACT), and ribosomal proteins (e.g., RPS5, RPS18, and RPL8). These genes are commonly used in insect studies [13,14,15], but their stability across different tissues, life stages, or environmental conditions is not consistent among species [16,17,18]. 

When it comes to bees, both RPS18 and RPL13A have been reported as suitable reference genes in the honey bee, *Apis mellifera* [15]. However, no reference genes have been validated for any bee as having uniform expression across different life stages. As with other insects, diapause is a special state where bees arrest development to adapt to extreme environmental conditions. It represents a distinct suite of developmental, behavioral, physiological, and biochemical attributes. During diapause, bee development is interrupted, and metabolic activity is suppressed; thus, diapause could result in dramatic changes in gene expression, as compared with other life stages of the bee [19,20,21]. Transcription analyses of the diapause to post-diapause transition identified 643 genes being upregulated and 242 genes being down-regulated in *M. rotundata* [19]. Other genes associated with metabolism, such as fatty acid biosynthesis, metal-binding juvenile hormone, innate immunity, and cold tolerance, were also substantially changed in diapausing *Drosophila montana* [21] and *Culex pipiens* [20].

*Megachile rotundata* overwinters as fifth (ultimate) instar larvae, but voltinism in this insect is variable. In any given location, and in any given year, part of the population will be univoltine, and part of the population will be bivoltine. The first eggs laid in a season complete development to the fifth instar mid-summer. After the fifth instar larvae have entirely consumed all the food provisions in their nest, they spin a cocoon. Larvae in the cocoon stage are referred to as prepupae. A portion of these will avert diapause, going on to pupate and complete development, making up the bivoltine segment of the population. The rest of the prepupae will go into diapause and overwinter, not pupating until the next spring. This life cycle provided an opportunity to compare gene expression for the same life stage, but in insects that went into diapause, and insects that averted diapause. Thus, we were able to compare the effects of having a diapause versus not having a diapause. We hypothesized that if genes are stably expressed before and after diapause as well as in diapausing and non-diapausing bees, they probably are also very stable across all other life stages. To evaluate the hypothesis, we selected 12 candidate reference genes utilizing information from a *M. rotundata* cDNA library (Table 1). Previously, we found expression of these genes was relatively stable for healthy and pathogen challenged bee larvae when the bees were exposed to different temperature conditions [6,8].

## 2. Materials and Methods

### 2.1. Source of M. rotundata Bees

We collected five individual *M. rotundata* from each of 11 life stages: eggs; 2nd, 3rd, 4th, and 5th instars; non-diapausing prepupae; diapausing prepupae; post-diapause pupae; pupae from non-diapause pre-pupae; adult males; and adult females. Some life stages were collected directly outdoors: eggs, all larval instars before cocoon formation, pupae from non-diapausing prepupae, adult males, and adult females. Those stages were all collected from leafcutting bee shelters in a farmer’s alfalfa seed field near Tremonton, Utah, during July and August 2011. Eggs are very difficult to distinguish from first instars because the latter remain in the egg chorion until they molt to the next instar. Thus, all references to eggs in this study might include first instars. Prepupae collected in the summer could contain a mix of diapausing and non-diapausing insects because no studies have been conducted to determine when diapause first occurs. Thus, for simplicity, we refer to summer collected prepupae as “non-diapausing prepupae.” 

We were able to distinguish pupae that originated from diapausing and non-diapausing prepupae. This is because pupae found in July and August originate from non-diapausing prepupae. Diapausing prepupae do not pupate in the summer, only in the spring, after at least three months exposure to cold temperature [22]. Diapausing prepupae were purchased in May as cocoons from a commercial bee supplier (JWM Leafcutter Inc., Nampa, ID, USA). The supplier collected these bees as cocoons the previous fall, then stored them at 4 °C for the winter (a standard beekeeping practice for *M. rotundata*). Non-diapausing prepupae would have died during cold storage. Thus, any live prepupae in this sample were in diapause. Post-diapause pupae were collected by incubating the diapausing prepupae at 39 °C for 7–10 days until they formed pupae. 

Each bee sampled was submerged in RNAlater (Life Technologies, NY, USA), soaked overnight (at 4 °C) using approximately 5x the estimated volume of the bee, and then was stored at −80 °C, still in the RNAlater, until processed (about six months). 

### 2.2. RNA Extraction 

Total RNA was isolated from individual bees using a PureLink™ Micro-to-Midi Total RNA Purification System (Life Technologies/Ambion, Grand Island, NY, USA). We isolated total RNA from 5 individuals for each life stage. The adult bees were ground individually under liquid nitrogen using a mortar and pestle. The frozen tissue powder was transferred to a 2.0 mL tube precooled with liquid nitrogen, and 1.2 mL RNA lysis solution added to each sample before the tissue thawed. Eggs, larvae, and pupae were individually placed directly into chilled, 2 mL microcentrifuge tubes with RNA lysis solution (0.6 mL for eggs and small larvae, 1.2 mL for pupae and large larvae). Each bee was homogenized independently by crushing it with a small plastic pestle, vigorously shaking it using a vortex mixer, then passing through an 18 gauge needle several times using an RNase-free syringe. Then, an equal volume of 70% ethanol was mixed into the sample. 

RNA was recovered from the lysate/ethanol mixture by filtering the sample through the RNA spin cartridge supplied with the kit. The flow-through was discarded, the cartridges were washed with buffer (as directed in the kit), and the total RNA was eluted by adding 30 μL of RNase-free water, incubating 1 min, and then centrifuging for 2 min at 13,000 rpm at room temperature. The elution steps were conducted three times for each spin cartridge, yielding approximately 90 μL of RNA solution. The concentration of total RNA in these samples was measured using a NanoDrop (Thermo Scientific, Inc. Wilmington, DE, USA), and the quality and integrity of total RNA were examined by electrophoresis. We only used samples where the ratios of A260/A280 were in the range of 2.0 to 2.2, and with typical two rRNA bands (28S and 18S), indicating that the total RNA had high quality and integrity [23]. The RNA samples were stored at −80 °C.

### 2.3. cDNA Synthesis

RNA expression levels were quantified using two-step RT-qPCR. First-strand cDNA was synthesized from the total RNA using a SuperScriptR VILO™ cDNA Synthesis Kit (Life Technologies/Invitrogen, Grand Island, NY, USA), as follows: 4 μL 5× VILO™ reaction mix and 2 μL 10× SuperScriptR enzyme were mixed with 2.5 μg total RNA in 14 μL DEPC-treated water, the samples incubated at 25 °C for 10 min followed by a second incubation at 42 °C for 60 min, after which the reactions were terminated by incubation at 85 °C for 5 min. The concentrations of the resulting cDNA samples were measured using a NanoDrop. The cDNA was stored at −20 °C until use. 

### 2.4. Primer Design

The second step, amplification of the cDNA, was conducted using primer pairs designed for 12 potential reference genes (Table 1). The gene sequences were obtained from our *M. rotundata* EST database (available in Genbank, http://www.ncbi.nlm.nih.gov/nucest/?term=Megachile+rotundata). The primers were designed according to the corresponding open reading frames for each of the conserved protein-coding domains within the candidate reference genes using a modified version of Primer3 software (http://fokker.wi.mit.edu/primer3/input.htm) [24]. Primers were designed using the following criteria: length (20 to 27-mer), melting temperature (60 to 64 °C), GC percentage (40 to 70%), an absence of significant secondary structures, and an amplicon length ranging from 64 to 108 bases (Table 1).

### 2.5. Quantitative Real-Time PCR

All RT-qPCR reactions were performed using Fast SYBR Green Master Mix kits (Life Technologies/AB Applied Biosystems, Grand Island, NY, USA) with 20 μL reactions consisting of 10 μL SYBR Green Master Mix, 0.4 μL of each primer (8 μM), 1 μL cDNA (400 ng/μL), and 8.2 μL water (Molecular Biology Grade, Thermo Scientific, Logan, UT, USA). Each primer pair was a separate reaction (no multiplex reactions). The PCR program was an initial 3 min denaturation at 95 °C, followed by 40 cycles of 15 s denaturation at 94 °C, 15 s annealing at 60 °C, and 15 s extension at 72 °C. Melting curves for the product were determined by raising the temperature from 55 to 90 °C in sequential steps of 1 °C for 2 s. Melting curves were evaluated to assure a single product was produced in each reaction. Any primer pairs that produced multiple peaks were discarded, and the results are not reported. 

Ct values were standardized by subtracting the baseline fluorescence, and then adjusting the output Ct based on the calculated PCR efficiency for each target cDNA using LinRegPCR (version 2012.0, Ruijter) [25]. Two reactions were run for each insect, always being conducted on different dates, and the resulting standardized Ct-values were averaged, yielding our reported Cq values. The replicate was five individually sampled insects for each of the 132 gene and insect stage combinations, for a total of 660 Cq values. We also included a no template cDNA negative control for every primer pair, also with a replicate of 5 conducted similarly to the insects (yielding another 60 Cq values). 

### 2.6. Data Analysis

Analysis of variance (ANOVA) was also used to evaluate whether the Cq values for RT-qPCR reactions with insect cDNA differed significantly from the blank controls for each primer pair and insect stage combination using Dunnett’s test. Student’s *t*-tests were carried out to compare gene transcripts of non-diapausing vs. diapausing prepupae, and pupae from non-diapausing prepupae vs. post-diapause pupae.

The expression stability across the different bee life stages was evaluated using both BestKeeper and NormFinder [26]. For the ANOVA method, the dependent variable was Cq values, and the main effects were the genes, life stages, and the interaction between these. This allowed for statistical comparison of the impact of the main effects on Cq values. We also ranked the stability based on the product of the average Cq and its standard deviation. Lower SD values in BestKeeper and lower SV values in NormFinder indicated lower variation in abundance in the different life stages, hence, greater stability of expression.

## 3. Results

### 3.1. Transcript Abundance Profiles for Different Genes and Life Stages

**Figure 1:**
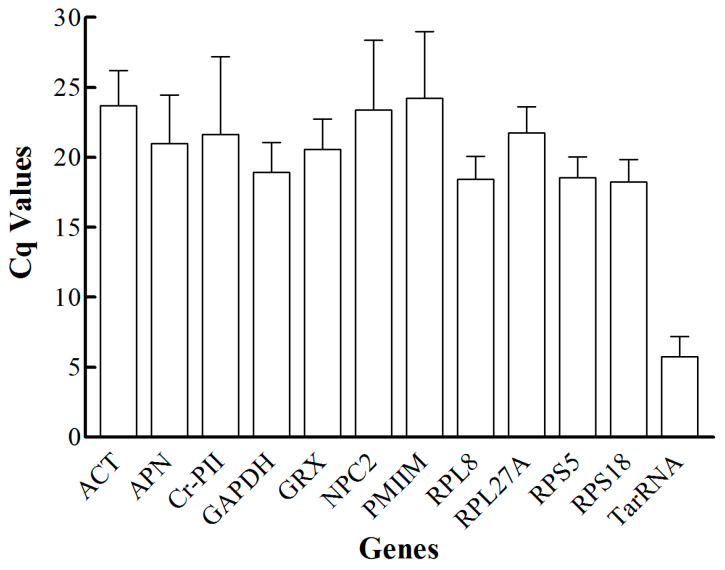
The RNA transcript abundance over all life stages of the alfalfa leafcutting bee, *Megachile rotundata*, for all the candidate reference genes. Cq values are normalized Ct values (units are polymerase chain reaction (PCR) cycle number).

Using the primer pairs in Table 1, RT-qPCR was used to evaluate gene expression levels of individual *M. rotundata* for each candidate reference gene. The bees sampled included different life stages collected at different times: eggs, larvae, diapausing and non-diapausing prepupae, and pupae from diapausing and non-diapausing prepupae, as well as female and male adults. Five bees were used for each life stage, and each RT-qPCR run was replicated twice for each bee. All Ct values were normalized to each other using LinRegPCR software to yield Cq values, as recommended by Ruijter et al. [25]. The highest level of transcript abundance among the candidate genes was observed for TarRNA, a nuclear gene encoding an antisense ribosomal RNA as it had the lowest Cq values, around 5–8 (Figure 1). The other transcripts tested had lower abundances with Cq values ranging from 15–30 (Figure 1).

### 3.2. Transcript Variation among Different Life Stages

**Figure 2:**
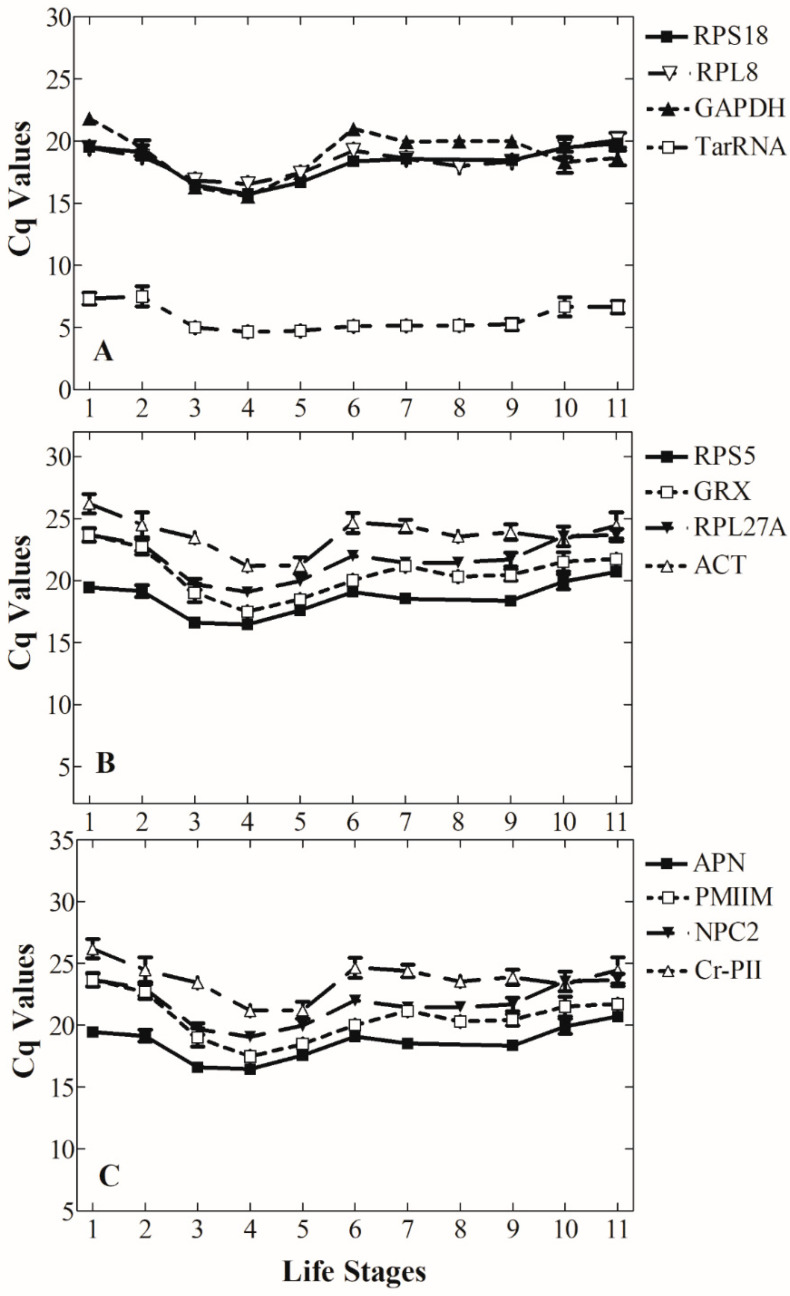
Variation in the abundance of the candidate reference gene transcripts over all life stages of *Megachile rotundata* expressed as Cq values for (**A**) genes with low variation in expression levels, (**B**) genes with intermediate variation in expression level, and (**C**) genes with the greatest variation in expression. Life stages are 1= Egg, 2 = 2nd instar, 3 = 3rd instar, 4 = 4th instar, 5 = 5th instar (prior to cocoon formation), 6 = non-diapausing prepupa, 7 = diapausing prepupa, 8 = post-diapause pupa, 9 = non-diapause pupa, 10 = Adult female, and 11 = Adult male.

Low variation of transcript level among different life stages is one of the most important criteria for determining the best reference gene. The Cq value for the TarRNA transcript was not significantly different among different larval and pupal stages (F = 1.11; d.f. = 7; *p* = 0.382) (Figure 2A and Table 2). The transcript abundance was uniform among the different immature life stages, except for the eggs and early instars, where transcript abundance was lower (Figure 2, Table 2). Transcript abundance for the RPL8, RPS5, RPS18, RPL27A, GRX, GAPDH, and ACT genes showed little variation among the egg, non-diapause pupae, post-diapause pupae, adult male, and adult female stages, but were variable during the larval life stages, as shown by the greater variation in Cq values. They also showed little variation in Cq values between diapausing prepupae and non-diapausing prepupae except for GRX and GAPDH (Figure 2A,B, Table 2). Cq values differed significantly across the different life stages for the other candidate genes: APN (F = 17.83; d.f. = 11; *p* < 0.001), PMIIM (F = 31.03; d.f. = 11; *p* < 0.001), NPC2 (F = 18.93; d.f. = 11; *p* < 0.001), and Cr-PII (F = 27.33; d.f. = 11; *p* < 0.001) (Figure 2C, Table 2). 

We used two additional statistical analyses, BestKeeper and NormFinder, to further evaluate and compare expression variability for the genes. Using these analyses, the RPS18 transcript had the most uniform abundance across life stages, among all the transcripts we evaluated, followed by RPL8 and GAPDH, which had the second to third lowest variability in both statistical analyses (Figure 2A, Table 3). The next most stable were the transcripts from the RPS5, GRX, RPL27A, TarRNA, and ACT genes, which ranked from 4th to 6th place (Figure 2A,B, Table 3). The transcripts from the other candidate genes APN, PMIIM, NPC2, and Cr-PII ranked as more variable (Figure 2C, Table 3). 

### 3.3. Stability of Gene Expression in Diapausing and Non-Diapausing Bees

**Figure 3:**
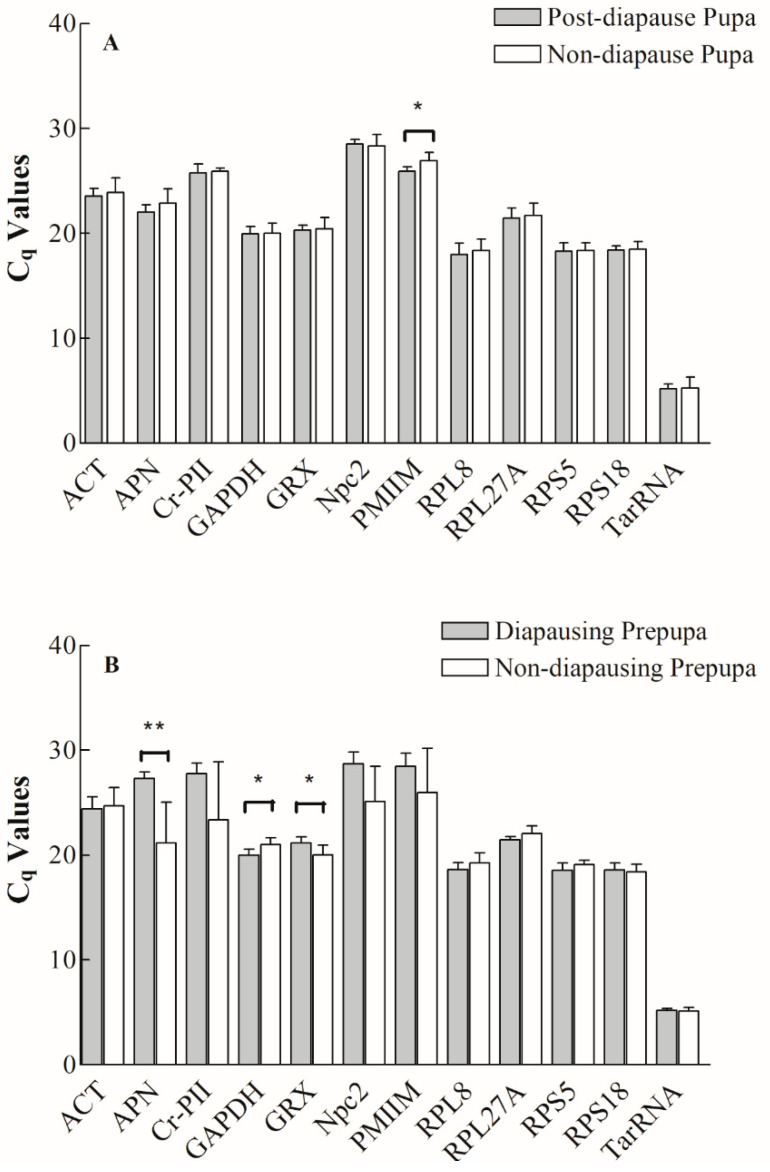
Comparison of twelve candidate reference gene transcripts of *Megachile rotundata* expressed as Cq values between post-diapause pupa vs. pupa from non-diapausing prepupa (**A**), and between diapausing prepupa vs. non-diapausing prepupa (**B**). Cq values are normalized Ct values. Treatments that are significantly different between diapausing and non-diapausing samples according to Student’s *t*-tests are labeled with asterisks (* *p* ≤ 0.05; ** *p* ≤ 0.01; *n* = 5). Error bars represent the standard deviation of the mean (SD).

Diapause is a common strategy for bees to overcome adverse environmental conditions. Thus, it may involve various gene expression changes, primarily decreased expression of metabolism and energy related genes and increased expression of cold resistance genes [19,20]. To test our hypothesis that genes stably expressed before and after diapause will also be stably expressed in all other life stages, we compared the twelve candidate reference genes between pupae from diapausing and non-diapausing prepupae, as well as between diapausing prepupae and non-diapausing prepupae (Figure 3). Only the PMIIM gene had significantly different expression levels between post-diapause pupae and pupae from non-diapause prepupae (t = 2.424; d.f. = 8; *p* = 0.042) (Figure 3A), while all other genes appeared to be stably expressed. For diapausing vs. non-diapausing prepupae, we found that expression varied significantly for APN (t = 3.504; d.f. = 8; *p* = 0.008), GAPDH (t = 2.523; d.f. = 8; *p* = 0.036), and GRX (t = 2.329; d.f. = 8; *p* = 0.048). In contrast, all other genes were expressed at similar levels in both diapausing prepupae and non-diapausing prepupae (Figure 3B).

## 4. Discussion

We found that genes stably expressed during diapause are generally stable across most or all development stages in *M. rotundata*. Not surprisingly, it is easier to find stable expression among various larval stages, or among adult insects, than across all life stages. Thus, one must remain cautious when selecting reference genes if the study includes insects across different life stages. 

Ribosomal protein genes were the least variable between diapausing and non-diapausing insects [21]. Therefore, these genes tend to be useful insect reference genes [27]. Among the four ribosomal protein genes we tested, RPL8, RPS5, and RPS18 proved to be better reference genes for *M. rotundata* than RPL27A, based on their lower variation across different life stages. We found RPS18 to be the best among all twelve of the candidate genes we tested. These results corroborate the use of RPS18 as a good reference gene for the honey bee [15], the Colorado potato beetle, the red flour beetle [16], the planthopper *Delphacodes kuscheli* [14], and the bed bug *Cimex lectularius* [28]. 

TarRNA is the transcribed antisense to the rRNA genes in the nuclear rDNA repeat region and was first identified in the yeast *Saccharomyces cerevisiae* [29]. Of all the genes we tested in *M. rotundata*, TarRNA had the most abundant transcript and had a stable expression level between diapause and non-diapause, and it was also fairly stable among the different life stages. The gene was ranked in fifth place. It is an acceptable reference gene if one is looking for something with high transcript abundance. However, we do not recommend it as one of the best reference genes due to its high abundance. It may not be a good normalizer for genes with low expression. We also found ACT to be an acceptable reference gene for *M. rotundata*. Although the transcript abundance for ACT was lower than for TarRNA, it was more variable across the different life stages than for both TarRNA and the ribosomal genes we tested. ACT has been extensively used as a reference gene in other insects including the honey bee, but its usefulness varied [14,15,30,31,32,33,34,35]. In particular, ACT was not a good reference gene in the silkworm or beet armyworm because transcript levels varied too greatly across different developmental stages [34].

## 5. Conclusions

We identified four genes for *M. rotundata* that are stably expressed across all life stages of the bee, and even during diapause. These genes may also prove useful as reference genes for other species of bees. Our top recommendations on reference genes for *M. rotundata* are RPS18 and RPL8, as these had the two most stable expression levels. RPS5 and RPL27A also are good reference genes, although expression levels are less stable than the top two genes. Although TarRNA was stable across most life stages, we do not recommend it because it had the most abundant transcript levels across all life stages. High abundance can make it difficult to normalize the baseline value in RT-qPCR data analysis accurately and may not be a good normalizer for weakly expressed genes. We found the abundance of the APN, PMIIM, NPC2, and Cr-PII gene transcripts were too variable among the life stages and do not recommend these be used if treatments include different life stages. 

## Figures and Tables

**Table 1:** Detailed information regarding the primers of 12 candidate genes.

Gene	Genbank Accession Number	Primer Sequences Forward and Reverse (5′ to 3′)	Amplicon Length	T_m_ (°C)	% GC	Coding Protein
ACT	SRX040740	CGGTTCGTGATAGGATTCATTGGTGGT	64	60.4	48.1	ß-actin
		TGGGAAGACATGTGTCATGTATGGGA		59.6	46.1	
APN	GD241616	TGCGCTGGACTGAGGAACGC	72	62.8	65.0	Aminopeptidase N
		GGCAAGCCAAGAGCGCGAAC		62.3	65.0	
Cr-PII	GD241657	TGGTCCGCAAGGTCGAAGCC	103	62.7	65.0	Cr-PII allergens
		GCGGAAGCCGGTTGGTGGAA		63.3	65.0	
GAPDH	GD240938	GTGCCGCCAAAGCTGTCGGT	81	63.6	65.0	Glyceraldehyde-3-phosphate dehydrogenase
		GCTGGTTTGCCAAGTCTGACCGT		62.1	56.5	
GRX	GD240911	ATTGGGCGACATGACCGGCG	76	63.2	65.0	Glutaredoxin (GRX) family
		AGCTTCTTCTGCAGCTCGCCA		61.6	57.1	
NPC2	GD241023	CGCACTCGTCCTGGTAGCGT	108	61.9	65.0	Niemann-pick type C2 (Npc2)
		CGTGCTTCGACATCGGTTCCCC		60.7	61.9	
PMIIM	GD241063	ACGCGACCGTCCATGTCTGTG	66	62.1	61.9	Peritrophic matrix insect intestinal mucin
		GTAAGCCGGTCCATCGGCCA		62.3	65.0	
RPL8	GD240977	GGAGCATCCTCACGGTGGTGG	71	62.5	66.6	Ribosomal protein L8
		TCCGGTACGACGAGCAGCAA		61.1	60.0	
RPL27A	GD240974	GGGGACTGCGATCAAGATGTCAAC	93	59.8	54.1	Ribosomal protein L27A
		GGTGGTGCAACCCACCAGCA		63.4	65.0	
RPS5	GD240987	CGGTCGCGCTGGTACTGTCA	64	62.1	65.0	Ribosomal protein S5
		ATGCGGCTTCCCTAGCACCG		62.6	65.0	
RPS18	GD240984	ACGAATTGGCAAGATGTCGCTCGT	91	61.2	50.0	Ribosomal protein S18
		GCATAACGGCGACCAACACC		59.3	60.0	
TarRNA	GD241002	GCGGTTAACGCCGCTGGGAA	80	63.3	65.0	Transcript antisense to ribosomal RNA
		CTACGGGCCTGGCACCCTCT		64.1	70.0	

**Table 2:** Comparison of stability of transcript abundance by NormFinder and BestKeeper among larval, prepupal, and adult stages of *Megachile rotundata*.

		Larval Stage				Prepupal Stage				Adult Stages			
Gene Name	Mean Rank	BestKeeper		NormFinder		BestKeeper		NormFinder		BestKeeper		NormFinder	
		SD ^1^	Rank	SV ^2^	Rank	SD	Rank	SV	Rank	SD	Rank	SV	Rank
RPS18	1	1.12	4	0.03	2	0.54	4	0.05	2	0.94	4	0.03	5
RPS5	2	1.04	3	0.05	5	0.52	3	0.07	10	0.83	2	0.02	1
RPL27A	3	1.35	5	0.02	1	0.44	2	0.06	5	1.06	6	0.04	6
GRX	4	1.80	11	0.04	4	0.74	7	0.02	1	0.92	3	0.03	3
RPL8	5	0.98	1	0.05	6	0.62	5	0.06	7	1.14	8	0.03	4
TarRNA	6	1.01	2	0.10	12	0.20	1	0.05	3	1.00	5	0.09	10
GAPDH	7	1.39	6	0.04	3	0.66	6	0.07	9	1.09	7	0.04	7
PMIIM	8	1.75	10	0.07	9	2.36	9	0.06	6	0.56	1	0.06	9
ACT	9	1.54	9	0.07	8	1.19	8	0.06	4	1.24	9	0.05	8
APN	10	1.41	7	0.06	7	3.53	12	0.12	12	1.25	10	0.03	2
NPC2	11	1.42	8	0.09	11	2.48	10	0.07	8	2.95	11	0.11	11
Cr-PII	12	2.30	12	0.08	10	3.36	11	0.11	11	3.66	12	0.15	12

^1^ SD (standard deviations): the ideal standard deviation should be less than 1 (Pfaffl et al., 2004); ^2^ SV (stability values): A low stability value indicates highly stable abundance of transcription for the gene (Pfaffl et al., 2004).

**Table 3:** Statistical analysis for stability of transcript abundance among different life stages of *Megachile rotundata*.

	Average	BestKeeper		NormFinder	
Gene	Rank	Rank	SD ^1^	Rank	SV ^2^
RPS18	1	4	1.22	2	0.06
RPL8	2	2	1.14	5	0.07
GAPDH	3	7	1.74	1	0.05
RPS5	4	3	1.17	6	0.07
GRX	4	6	1.55	3	0.06
RPL27A	4	5	1.48	4	0.06
TarRNA	5	1	1.00	10	0.14
ACT	6	8	1.53	8	0.08
APN	7	9	2.79	7	0.07
PMIIM	8	10	4.26	9	0.12
NPC2	9	11	4.54	11	0.14
Cr-PII	10	12	5.00	12	0.18

^1^ SD (standard deviations): the ideal standard deviation should be less than or close to 1 (Pfaffl et al., 2004); ^2^ SV (stability values): A low stability value indicates highly stable levels of transcription across the life stages (Pfaffl et al., 2004).

## Data Availability

Sequence data from this article can be found in GenBank (http://www.ncbi.nlm.nih.gov/nucest/?term=Megachile+rotundata) under accession numbers GD240906 to GD241784 and GE342618 to GE342638.
